# A model for predicting celiac disease among undiagnosed subjects established with data from the HUNT4 study

**DOI:** 10.1186/s12876-026-04837-y

**Published:** 2026-04-16

**Authors:** Mohammad Sayeef Alam, Rebecka Hjort, Iris H. Jonkers, Sebo Withoff, Kristian Hveem, Knut E. A. Lundin, Ludvig M. Sollid, Eivind Ness-Jensen

**Affiliations:** 1 Department of Public Health and Nursing, HUNT Center for Molecular and Clinical Epidemiology, NTNU, Norwegian University of Science and Technology, Trondheim, Norway; 2 HUNT Research Centre, NTNU, Norwegian University of Science and Technology, Levanger, Norway; 3 Department of Medicine, Levanger Hospital, Nord-Trøndelag Hospital Trust, Levanger, Norway; 4 Department of Genetics, University Medical Center Groningen, University of Groningen, Groningen, Netherlands; 5 Department of Research, St. Olav’s hospital, Trondheim, Norway; 6 Department of Gastroenterology, Oslo University Hospital - Rikshospitalet, Oslo, Norway; 7 Norwegian Coeliac Disease Research Centre, Institute of Clinical Medicine, University of Oslo, Oslo, Norway; 8 Department of Immunology, Oslo University Hospital - Rikshospitalet, Oslo, Norway; 9 Department of Molecular Medicine and Surgery, Karolinska Institutet and Karolinska University Hospital, Stockholm, Sweden

**Keywords:** Elastic-net regression, Polygenic risk score, Decision curve analysis, Population-based screening, Celiac disease, Risk stratification

## Abstract

**Introduction:**

Celiac disease (CeD) is an immune mediated disorder with substantial underdiagnosis, particularly in adults with non-specific symptoms. Universal population screening remains under debate due to cost-effectiveness and potential quality-of-life implications. Clinical prediction tools aid clinicians in identifying individuals who might benefit from targeted screening.

**Methods:**

Using data from 52,000 adults in the fourth population-based Trøndelag Health Study (HUNT4) in Norway, we developed and internally validated a non-diagnostic prediction model for previously undiagnosed CeD. The model incorporated predictors spanning genetics, lifestyle, symptoms, comorbidity, and biomarkers. Elastic-net logistic regression with nested cross-validation was chosen due to the low disease prevalence and imbalanced case-control distribution. Model performance was assessed by the area under the receiver operating characteristic curve, area under precision-recall curve, and calibration plots. Clinical utility was evaluated at different risk thresholds.

**Results:**

Among 465 newly diagnosed cases and 51,515 controls, polygenic risk score emerged as the strongest predictor for CeD, alongside diabetic history, kidney function, chronic discomfort, and perceived health status. The model achieved high discrimination (84%) and good calibration (0.009). The model outperformed universal population screening by detecting more cases, especially when the predicted risk was less than 2%. Increasing sample prevalence for sensitivity analysis, revealed that the model was better in population scenarios compared to high-risk/high-prevalence scenarios.

**Conclusion:**

The multifactorial model demonstrated strong potential for guiding targeted screening for CeD, especially in low-prevalence, true population-based settings. While not a diagnostic tool, it may support clinical decision-making by identifying individuals at elevated risk. External validation and cost-effectiveness studies are needed before it can be implemented in primary care.

**Supplementary Information:**

The online version contains supplementary material available at 10.1186/s12876-026-04837-y.

## Introduction

Celiac disease (CeD) is chronic immune-mediated disorder with a global prevalence of 1–2% [1, 2]. It can develop in genetically susceptible individuals who are exposed to gluten, a common component of the storage proteins of wheat, rye, and barley [3]. This exposure triggers a systemic inflammation, with small intestinal inflammation and villous blunting as a hallmark. The inflammation might lead to a wide range of symptoms and findings, including fatigue, stunting, anemia, osteoporosis, infertility, and in rare cases even cancer [4, 5].

Due to the varying severity and symptoms, CeD often times is either misdiagnosed or remains completely undiagnosed, which led to the “celiac iceberg” analogy [6, 7]. It has been reported that for every individual that is diagnosed around 4 to 7 individuals are likely to remain undiagnosed [8]. However the burden of previously undiagnosed cases is relatively lower in Nord Trøndelag county, Norway, with 1.2 new, previously undiagnosed cases per previously diagnosed case, but still quite high with ~ 55% of the cases being undiagnosed [9, 10]. Once diagnosed the current treatment option is limited to strict lifelong adherence to a gluten free diet (GFD), which has shown to improve the cumulative damage suffered by the gut and symptom remission [11].

Even with most of the cases remaining undiagnosed, it is widely debated whether to perform universal population screening. Firstly the cost-effectiveness of population screening is yet to be proven [12], and secondly, the negatively impact of gluten-free diet on the individuals’ quality of life as it would mean lifelong dietary restriction [13, 14]. Singh and colleagues [15], recommend screening those presenting certain symptoms such as diarrhea, comorbidity (diabetes mellitus, thyroid disease, and osteoporosis among others), and/or relatives of diagnosed individuals.

Prediction models could offer a promising approach to improving early detection of CeD by targeted screening, also in population-based settings, where individuals remain undiagnosed and symptoms are often non-specific. Existing prediction studies have relied upon previously diagnosed cases, high risk families, and individuals presenting certain symptoms, and have been mostly limited to pediatric cohorts [16–19]. These approaches could help in a highly selected context, however, fail to capture asymptomatic or atypical cases often seen among adults.

In the current study, we propose a model to address these limitations by incorporating a comprehensive set of predictors, ranging from genetics to lifestyle, symptoms, comorbidities and biomarkers, allowing the clinician to take a decision on whether to offer serological testing or not. The current model has been developed to reflect the real-world scenarios where the prevalence is quite low, and distribution of cases and non-cases are highly disproportionate.

## Methods

### Data source

The Trøndelag Health Study (HUNT) is a longitudinal study conducted in Trøndelag County of Norway based on repeated health surveys of the general population since the 1980ies [20]. The current study population is derived from the fourth round of the study, HUNT4, conducted in 2017–2019 [21]. All adults (> 20 years) of the former Nord-Trøndelag County were invited to participate and corresponding serum samples were stored in HUNT Biobank [22]. A total of 56,042 (54%) individuals participated, including 30,598 women (54.5%) and 25,444 (45.5%) men.

### Celiac disease participants

Serum samples collected from all participants were analyzed for transglutaminase 2 (TG2) immunoglobulin (Ig) A and IgG antibodies using a previously described assay [23]. Individuals with a positive screening were called in for endoscopic biopsy confirmation. A diagnosis of CeD was established if duodenal biopsies revealed mucosal damage corresponding to Marsh grade 3. The screening identified new, previously undiagnosed participants with CeD. In addition, seronegative participants with known, previously diagnosed and treated CeD were identified from medical journal searches. Remaining seronegatives, were considered as controls and were not biopsied. More details about the identification of celiac participants in HUNT4 can be found in a previous study [24].

### Study design

A case-control study design was used, with the new, previously undiagnosed participants as cases and the remaining seronegative HUNT4 participants as controls, excluding the previously known cases. The individuals were divided into train and validation sets (70:30) stratified by the outcome to maintain similar prevalence level in both sets.

### Predictors

The participants of the study were asked to fill in patient-reported outcome measures on Norwegian version of Short Form 36 Health Survey Questionnaire, Chalder Fatigue Scale, Hospital Anxiety and Depression Scale, Gastrointestinal Symptom Rating Scale-Irritable Bowel Syndrome, Coeliac Disease Symptom Index, Coeliac Disease Assessment Questionnaire and five in-house made questions about diet [25–30]. Additionally, information on demography, lifestyle factors, symptoms, comorbidities and corresponding medicine intake, and biomarkers were collected in the HUNT4 survey and considered in the analysis. Except for the biomarkers, all responses were self-reported. The inclusion of the predictors was based on a prior assessment of their respective relevance to CeD based on existing literature and research interest. A total of 66 variables were recorded and regrouped into 29 predictors; details are shown in Supplementary Table 1.

#### Polygenic risk score

We reproduced a previously developed and validated polygenetic risk score (PRS) consisting of 1,661 SNPs, which were originally selected using a penalized regression approach. Details of the PRS are available in a prior publication [31]. The PRS was calculated in our dataset using PLINK, as the sum of risk alleles weighted by their effect size from a large-scale GWAS [31, 32]. We excluded SNPs with poor imputation quality (r^2^ < 0.7), resulting in the removal of 79 SNPs (4.8%) from the original set prior to computing the PRS in the current cohort [33].

#### Lifestyle factors

Bread consumption was considered as a proxy for gluten intake. Smoking has been found to be negatively associated with CeD in previous studies [34]. Exercise, alcohol consumption, and body mass index (BM) were considered as predictors based on findings from previous studies [35–37].

#### Symptoms

The HUNT study provided information on several symptoms. General health status, chronic discomfort, muscular and joint pain, fatigue, headache, mental distress including anxiety and depression, and overall satisfaction with life were all categorized and included. Gastrointestinal symptoms such as diarrhea, stomach pain, heartburn, constipation, bloating, and corresponding medication intake were all categorized under gastrointestinal problems and included.

#### Comorbidity

We considered self-reported cancer, cardiovascular disease including stroke, myocardial infarction and angina pectoris, diabetes mellitus, thyroid disease including gout, and spondylitis and rheumatoid arthritis in the modelling. Clinical biochemistry results were cross-checked for only diabetes and thyroid disease.

#### Biomarkers

Estimated glomerular filtration rate (eGFR), cholesterol, high density lipoprotein (HDL), triglycerides, C-reactive protein (CRP), hemoglobin, and thyroid stimulating hormone (TSH) were all measured and considered within the model. Thresholds were decided based on laboratory handbook of Helse Nord-Trøndelag [38], except for hemoglobin whose range was provided by the HUNT Databank [22].

### Statistical analysis

We performed descriptive analysis of all variables and tested the statistical difference between cases and controls using the Welch’s two sample t-test for normally distributed continuous variables and Peason’s chi-squared test for categorical variables. The proportion of missing data ranged from 0% to nearly 20%. The missing data were imputed using the “mice” package in R [39].

#### Model selection

To prevent optimism bias and data leakage, the entire preprocessing and model-selection pipeline was nested within each outer fold, we performed imputation, standardization and then ran elasticnet based variable selection, and finally hyperparameter tuning exclusively inside the inner cross-validation (CV). The optimal inner-CV was then refitted onto the outer training split and evaluated once on its held-out outer-validation split.

Elastic-net logistic regression was employed for prediction modelling, which combines both L1 (lasso) and L2 (ridge) penalties to perform simultaneous variable selection and coefficient shrinkage [40]. The elastic-net approach is controlled by two hyperparameters: the penalty parameter (lambda) and mixing parameter (alpha), where alpha ranges from 0 (pure ridge regression) to 1 (pure lasso regression). To identify optimal hyperparameters, we implemented a nested cross-validation strategy with three outer folds and three inner folds. Within each outer fold, we evaluated 330 combinations of hyperparameters (30 lambda values logarithmically spaced between $\:{10}^{-5}$ and 10, and 11 alpha values from 0 to 1 in increments of 0.1). The combination yielding the highest area under the receiver operating characteristic curve (AUROC) in the inner cross-validation was selected. Final hyperparameter values were determined by taking the geometric mean of lambda and median of alpha across the outer folds.

#### Model estimation

Following hyperparameter optimization, we fitted the final elastic-net logistic regression model on the complete training dataset using the selected lambda and alpha values. All predictor variables underwent standardized preprocessing, dummy encoding of categorical predictors, removal of zero-variance predictors, and normalization of all numeric predictors. The model was trained without interaction terms to maintain interpretability and reduce overfitting risk. And all preprocessing steps were estimated on the training portion only and applied to the corresponding validation poration within each outer fold to avoid information leakage.

#### Model performance

The model performance was evaluated comprehensively on both training and validation datasets using measures of discrimination, calibration and overall fit. Discrimination, reflecting the model’s ability to distinguish between cases and controls, was quantified using the AUROC. Agreement between the predicted probability of the outcome against the actual observed outcome was assessed through calibration plots and Brier score, with a lower score indicating better agreement. We also calculated the Nagelkerke R^2^ to quantify the proportion of variance explained by the model. Bootstrap resampling with 2000 iterations was used to derive 95% confidence intervals (CIs) for all performance metrics in the test set, accounting for sampling variability in our estimates.

#### Validation

The prediction was then made for the individuals from the validation set using the model developed based on the nested cross-validated training dataset. The model performance statistics were calculated as described above. In addition, we present the 95% CIs for each metric using bootstrap resampling.

#### Clinical usefulness

To assess the clinical utility of the prediction model, we evaluated its performance across multiple predicted CeD risk thresholds. These predicted risk thresholds were determined based on positive predictive value (PPV) in the training set. Since the prevalence of previously undiagnosed CeD was only 1% in the population, chance of the detecting a true positive case also diminishes. Hence predicted risk was determined at different (1%, 1.5%, 2%, 5%, and 10%) PPV thresholds. For each threshold we computed 95% CIs using bootstrap resampling to quantify uncertainty in these performance estimates.

#### Sensitivity analysis

To assess the robustness across different prevalence scenarios, we conducted a prevalence sensitivity analysis by systematically varying the proportion of cases to controls in the test set. We maintained all cases (*n* = 465) and randomly down sampled the control group to achieve target prevalence of 1%, 2.5%, 5%, and 10%. For each prevalence level, we re-evaluated model discrimination using AUROC and area under precision-recall curve (PR-AUC) metrics. Additionally, we assess thresholds-specific performance metrics, namely, sensitivity, specificity, PPV and negative predictive value (NPV). Decision curve analysis was performed at each prevalence level to evaluate clinical utility across different risk thresholds. Bootstrap resampling with 2000 iteration was used to compute 95% CIs for all performance metrics at each prevalence scenario. This analysis allowed us to evaluate whether model performance remained stable when applied to populations with different underlying disease prevalence which is critical for assessing generalizability to clinical settings with varying case control ratios.

## Results

### Study participants

In total, 1,107 HUNT4 participants (2%) had a positive serological screening and were invited for biopsy testing. Among these, 465 were confirmed as new, previously undiagnosed cases, corresponding to about 1% population. Additionally, 361 previously known individuals with CeD were identified through their medical journal record searches. This left 51,515 non-cases included as controls. The descriptive statistics for the cases and controls are presented in Table 1. Newly diagnosed cases were slightly younger than the non-cases (mean age 51 vs. 55 years respectively) and were more often females (54%), but the sex ratio was similar between cases and non-cases. The mean PRS estimates were 1.7 standard deviation units more among cases than controls. Newly diagnosed cases had high education, lower weight and smoked less than non-cases. They reported better health status and life satisfaction and less discomfort, pain and mental health problems. They also had lower rates of cardiovascular disease and diabetes mellitus better kidney function and less inflammation based on CRP.

**Table 1:** Baseline characteristics of celiac disease cases and controls

Variable	Cases *N* = 465^1^	Controls *N* = 51,515^1^
Sex		
Male	214 (46.0%)	23,401 (45.4%)
Female	251 (54.0%)	28,114 (54.6%)
Age (years)	51.33 (16.37)	55.17 (17.57)
Polygenic Risk Score	1.20 (0.86)	0 (1)
Education Level		
< 13 years	253 (54.6%)	30,874 (61.0%)
>= 13 years	210 (45.4%)	19,739 (39.0%)
Missing	2 (0.4%)	902 (1.8%)
Municipality		
Fjord	325 (69.9%)	35,008 (68.0%)
Inland	63 (13.5%)	6,578 (12.8%)
Coast	77 (16.6%)	9,922 (19.3%)
Missing	0	7 (< 0.1%)
BMI Category		
Underweight	7 (1.5%)	421 (0.8%)
Normal weight	180 (39.0%)	16,250 (32.4%)
Overweight	177 (38.4%)	21,202 (42.2%)
Obesity	97 (21.0%)	12,317 (24.5%)
Missing	4 (0.9%)	1,325 (2.6%)
Smoking Status		
Never	236 (50.9%)	21,993 (43.4%)
Ever	228 (49.1%)	28,665 (56.6%)
Missing	1 (0.2%)	857 (1.7%)
Exercise Frequency		
Never-Moderate	165 (35.8%)	17,822 (35.6%)
High	296 (64.2%)	32,191 (64.4%)
Missing	4 (0.9%)	1,502 (2.9%)
Alcohol Frequency		
Never	33 (7.2%)	5,547 (11.0%)
Moderate	342 (74.2%)	34,589 (68.9%)
High	86 (18.7%)	10,075 (20.1%)
Missing	4 (0.9%)	1,304 (2.5%)
Bread Consumption		
< 3 per day	295 (75.8%)	30,781 (77.0%)
>=3 per day	94 (24.2%)	9,193 (23.0%)
Missing	76 (16%)	11,541 (22%)
Self-Rated Health Status		
Poor/Not good	73 (15.8%)	12,018 (23.9%)
Good/Very good	388 (84.2%)	38,189 (76.1%)
Missing	4 (0.9%)	1,308 (2.5%)
Chronic Discomfort	152 (33.0%)	21,950 (43.6%)
Missing	5 (1.1%)	1,158 (2.2%)
Physical Pain	382 (86.0%)	42,617 (88.7%)
Missing	21 (4.5%)	3,467 (6.7%)
Mental Health Problems	232 (63.9%)	28,433 (73.0%)
Missing	102 (22%)	12,574 (24%)
Life Satisfaction		
High	269 (58.5%)	26,617 (52.9%)
Low-Moderate	191 (41.5%)	23,689 (47.1%)
Missing	5 (1.1%)	1,209 (2.3%)
Gastrointestinal Complaints		
Never	46 (11.9%)	5,455 (13.5%)
Yes	342 (88.1%)	34,987 (86.5%)
Missing	77 (17%)	11,073 (21%)
History of Cancer	30 (6.6%)	3,983 (8.1%)
Missing	8 (1.7%)	2,630 (5.1%)
Cardiovascular Disease	113 (24.8%)	14,953 (30.4%)
Missing	10 (2.2%)	2,313 (4.5%)
Diabetes	22 (4.8%)	4,905 (9.9%)
Missing	8 (1.7%)	1,812 (3.5%)
Thyroid Disease	56 (12.3%)	7,074 (14.5%)
Missing	11 (2.4%)	2,875 (5.6%)
Connective Tissue Disease	39 (8.6%)	4,914 (10.2%)
Missing	14 (3.0%)	3,357 (6.5%)
eGFR Category		
Normal	310 (66.7%)	28,568 (55.5%)
Reduced	155 (33.3%)	22,923 (44.5%)
Missing	0 (0%)	24 (< 0.1%)
Lipids Category		
Normal	173 (37.2%)	17,756 (34.5%)
Abnormal	292 (62.8%)	33,738 (65.5%)
Missing	0 (0%)	21 (< 0.1%)
CRP Category		
Normal	389 (83.7%)	39,980 (77.7%)
High	76 (16.3%)	11,479 (22.3%)
Missing	0 (0%)	56 (0.1%)
Haemoglobin Category		
Normal	456 (98.3%)	49,879 (97.2%)
Anemia	8 (1.7%)	1,448 (2.8%)
Missing	1 (0.2%)	188 (0.4%)

Physical pain included joint pain, muscular pain, headache, including migraine, and medicine for physical pain; Mental problems included fatigue, anxiety, depression, memory problems, symptoms of anxiety or depression, and medicine for anxiety or depression; Gastrointestinal complaints included stomach pain, nausea, diarrhea, constipation, bloating, heartburn, and medication for heartburn and constipation; Cardiovascular disease included self-reported atrial fibrillation, myocardial infarctions, heart failure, angina, high blood pressure, stroke, and medicine for high blood pressure; Diabetes mellitus included self-reported diabetes status and hyperglycemia and high HbA1c values; Thyroid disease included self-reported hypothyroidism, hyperthyroidism, medication for thyroid disease, and pathological TSH values; Connected tissue disease included self-reported rheumatoid arthritis, gout, and Bechterew disease

*BMI* Body mass index, *eGFR* Estimated glomerular filtration rate, *CRP* C-reactive protein, *TSH* Thyroid stimulating hormone

^1^n (%); Mean (SD), Percentage missing (out of total N)

The missingness was associated with the observed participation characteristics (not shown in Table) and supported treating missing data as missing at random (MAR) and conditioning the imputation models on observed variables.

### Model specification and performance

During the nested cross-validation process, the optimal settings were a small penalty for shrinking coefficient (lambda = 0.0016), keeping most values close to their original estimate, and a strong tendency to select most important variables (alpha = 0.8), prioritizing removing less important ones. The elastic-net logistic regression selected five predictor variables to be included in the final model (Supplementary Table 2). In the current cohort, the PRS was associated with 3.6-fold higher odds of celiac disease, whereas diabetes mellitus, eGFR, health status, and chronic discomfort were associated with 47%, 33% and 32%, and 22% lower odds respectively.

**Fig. 1:**
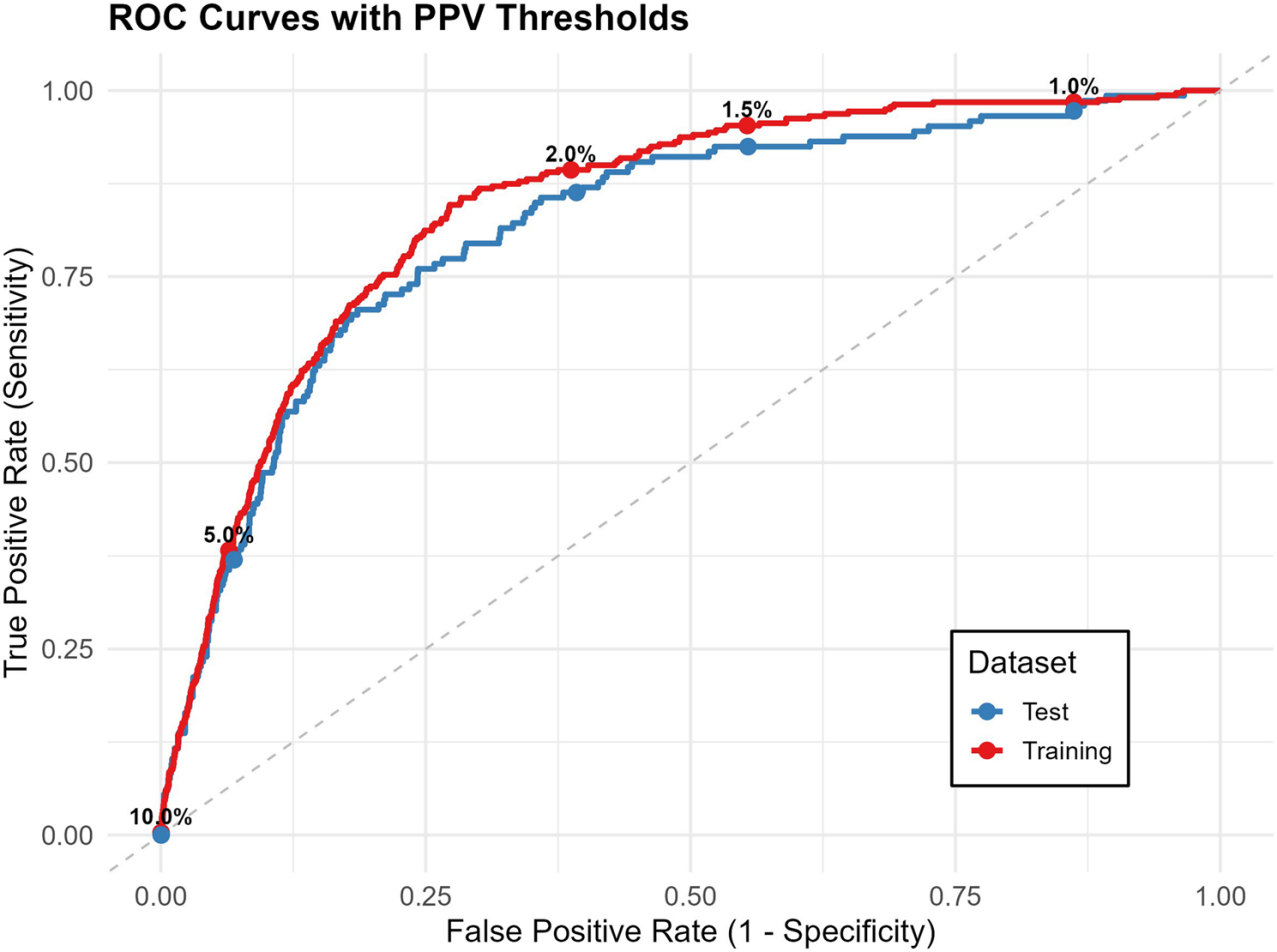
Receiver operating characteristic curves for discrimination between cases and controls in the training and test datasets

The final trained model demonstrated good discrimination (84%) in the training data set (Fig. 1). The PR-AUC was quite low in the training data set (4.6%); however, this is common in low prevalence settings [41]. The model was able to explain 14.1% of the variation within the training dataset, which indicates that the remaining variation arises from unaccounted/removed variables. The numerical estimates and their corresponding 95% CIs are reported in Table 2.

**Table 2:** Model performance estimates with 95% confidence intervals in the train and test sets

Metric	Training	Test
AUROC	0.841 (0.821–0.860)	0.816 (0.778–0.848)
PR-AUC	0.046 (0.038–0.061)	0.044 (0.033–0.063)
Brier Score	0.009 (0.008–0.009)	0.009 (0.009–0.009)
Nagelkerke R²	0.141 (0.124–0.158)	0.126 (0.096–0.155)

*AUROC* Area under receiver operating characteristic curve, *PR-AUC* Area under precision-recall curve

### Validation

The model performance in the test data set was comparable to its training counterpart (Supplementary Table 3). The AUROC remains quite high at 81%. The explained variation by the model in the training data set dropped to 12.6% on the testing data set. The PR-AUC and Brier score were the same as observed in the training dataset (Table 2). The PR-AUC and calibration plots are illustrated in Supplementary Figs. 1 and 2, respectively.

### Clinical usefulness

The decision curve analysis indicated that in the ~ 1% prevalence setting of HUNT4, the model yields a higher net benefit than both “screen-all” and “screen-none” at low decision thresholds, approximately 0.2%-~1% predicted risk. In the current cohort, population screening detected 1.2 newly diagnosed case per previously known case, leading to a sensitivity of 54.5%. The model achieved a sensitivity higher than the one observed by population screening but at lower predicted risk thresholds. When the predicted risk threshold was set to achieve a PPV threshold of 2%, the model correctly identifies 89.3% (85.9–92.8%) cases, corresponding to 10.7% missed. The cases missed further drops to 1.6% when the predicted risk threshold was set to achieve 1% PPV in the training dataset. In the testing dataset only 7.5% and 2.7% of the cases were missed at respective corresponding thresholds (Table 3).

**Table 3:** Distribution of missed individuals based on the risk

Dataset	Threshold	Sensitivity	Specificity	PPV	NPV	TP	FP	TN	FN	Cases missed, %
Train	0.002	0.984 (0.969–0.997)	0.138 (0.135–0.142)	0.010 (0.010–0.010)	0.999 (0.998-1.000)	314	31,086	4981	5	1.6%
	0.004	0.953 (0.928–0.975)	0.447 (0.441–0.452)	0.015 (0.015–0.015)	0.999 (0.999-1.000)	304	19,962	16,105	15	4.7%
	0.007	0.893 (0.859–0.928)	0.613 (0.607–0.618)	0.020 (0.019–0.021)	0.998 (0.998–0.999)	285	13,965	22,102	34	10.7%
	0.032	0.232 (0.188–0.279)	0.961 (0.959–0.963)	0.050 (0.041–0.060)	0.993 (0.993–0.993)	74	1406	34,661	245	76.8%
	0.167	0.003 (0.000-0.009)	1.000 (1.000–1.000)	0.100 (0.000-0.333)	0.991 (0.991–0.991)	1	9	36,058	318	99.7%
Test	0.002	0.973 (0.945–0.993)	0.140 (0.135–0.146)	0.011 (0.010–0.011)	0.998 (0.996-1.000)	142	13,281	2167	4	2.7%
	0.004	0.925 (0.877–0.966)	0.446 (0.438–0.454)	0.016 (0.015–0.016)	0.998 (0.997–0.999)	135	8556	6892	11	7.5%
	0.007	0.863 (0.808–0.911)	0.607 (0.600-0.615)	0.020 (0.019–0.022)	0.998 (0.997–0.999)	126	6068	9380	20	13.7%
	0.032	0.240 (0.171–0.315)	0.957 (0.954–0.960)	0.050 (0.036–0.066)	0.993 (0.992–0.993)	35	659	14,789	111	76%
	0.167	0.000 (0.000–0.000)	1.000 (0.999-1.000)	0.000 (0.000–0.000)	0.991 (0.991–0.991)	0	4	15,444	146	100%

### Sensitivity

While increasing the prevalence levels from 1% to 10% did not change the discriminatory ability of the model and remained stable at ~ 80%, substantial improvement in PR-AUC from 5% to 35% was observed (Supplementary Table 4). The decision curve analysis illustrated in Supplementary Fig. 3 presents the net benefit of calling patients for serological screening. At low prevalence, such as routine primary care, or regions with relatively lower prevalence, calling individuals based on their predicted probability yields more net benefit than population screening, however, with increasing prevalence, such as among first degree relatives or population with existing autoimmune disease, population screening carries more net benefit.

## Discussion

In the current study we developed and internally validated a clinical prediction model to assist in identifying previously undiagnosed cases of CeD in a population-based setting. The model detected more cases than captured through universal population screening. In addition to the polygenic risk score, the elastic-net regression selected four non-genetic non-classical predictors reduced eGFR, poor self-rated health, chronic discomfort, and diabetes mellitus. These variables were inversely associated with undiagnosed CeD, which is counterintuitive, however in population screening, the new cases often have early or subclinical presentation. The younger age of the case group, along with participation of healthier individuals in survey and follow-up could also contribute to such observations. Composite gastrointestinal symptoms, thyroid disease, anemia and other lifestyle variables did not improve discrimination and were excluded. This suggests that, in adult population-based settings, non-classical signals related to overall health and comorbidity may help identify candidates for serological testing. The model demonstrated strong discriminatory ability and good calibration, suggesting robust performance in distinguishing the new previously undiagnosed CeD cases from disease free individuals. The model was developed to be useful in real-world population settings, rather than targeting high-risk individuals.

To evaluate clinical usefulness, we assessed model performance across a range of risk thresholds. With the increasing range of predicted risk the advantage of net benefit starts decreasing, which is consistent with the increasing trade-off between false-positive and missed cases in low-prevalence context. This tradeoff between sensitivity and PPV is consistent with findings from a UK based model and underscores the challenge of balancing case detection with clinical utility especially in low prevalence settings [18]. The current model is best suited for population cohorts, but less useful in high-risk population groups. In our low-prevalence population where PPV is strongly prevalence-dependent, lower decision thresholds yielded higher case detection with positive net benefit relative to population screening, whereas in higher-prevalence contexts the advantage of targeted screening diminished.

Our study has several strengths. We used a large, representative population cohort with comprehensive phenotypic and genotypic data. Predictor selection was informed by a priory knowledge from clinical experience and relevant literature, and the model was developed using nested cross validation to ensure generalizability. The integration of genetic risk with real world clinical and lifestyle data enhances the model’s applicability in primary care, especially where CeD prevalence is low and underdiagnosis is common.

The study also has several limitations. The model was developed and validated within the same population cohort, and external validation is needed to assess generalizability. Self-reported symptoms and comorbidities may be subject to bias and misreporting. In addition, as the biopsy was only undertaken by seropositive cases, some non-cases may include undiagnosed CeD as well, introducing differential verification bias, which might attenuate associations and underestimate model performance. Due to the low prevalence of CeD, increasing the folds in cross-validation would result in each fold having a smaller number of cases, thereby making it difficult for the model by introducing more noise. Although missing data were imputed using robust methods, residual bias cannot be ruled out. Additionally, while PRS offers a powerful predictive signal it may not capture all genetic risk factors, particularly since many of the HLA variants were removed in the current PRS. The apparent opposite directional unpenalized odds ratio arise due to the imbalance in the baseline characteristics, where the proportion of certain comorbidities was higher among the controls, thereby attenuating the expected association. As the prevalence of previously undiagnosed CeD is very low (1%), even decent clinical model using real clinical data would expect low PPV and generate many false positive. This might lead to unnecessary follow-up test and increased healthcare cost.

Methodologically, the nested cross-validation and prevalence sensitivity analysis allowed us to assess model stability across different case-control ratios. Compared to existing genetic models that rely on HLA typing [42–44], our PRS based approach is more scalable and accessible for integration into clinical workflows.

To conclude, in clinical practice, this model could support targeted screening for CeD, particularly in individuals with elevated overall genetic risk or relevant symptoms. While it does not replace diagnostic testing, it may assist clinicians in deciding when to offer serological testing. Future research should focus on prospective validation in diverse cohorts, on cost effectiveness, and integration into population health registry portals to facilitate real-time decision support. By improving case detection in low prevalent populations, such tools may help reduce the burden of undiagnosed CeD and enable timely dietary intervention.

## Supplementary Information

Supplementary Material 1.

Supplementary Material 2.

## Data Availability

The Trøndelag Health Study (HUNT) has invited individuals aged 13+ years to four surveys between 1984 and 2019. Comprehensive data from more than 140,000 individuals having participated at least once and biological material from 78,000 individuals are collected. The data are stored in HUNT databank and biological material in HUNT biobank. HUNT Research Centre has permission from the Norwegian Data Inspectorate to store and handle these data. The key identification in the database is the personal identification number given to all Norwegians at birth or immigration, whilst de-identified data are sent to researchers upon approval of a research protocol by the Regional Ethical Committee and HUNT Research Centre. To protect participants’ privacy, HUNT Research Centre aims to limit storage of data outside HUNT databank and cannot deposit data in open repositories. HUNT databank has precise information on all data exported to different projects and are able to reproduce these on request. There are no restrictions regarding data export given approval of applications to HUNT Research Centre. The codes supporting the current study have not been deposited in a public repository because the pipeline is under construction but are available from the corresponding author on request. For more information see: ( http:/www.ntnu.edu/hunt/data ).
