# Supplementary material for: A model for predicting celiac disease among undiagnosed subjects established with data from the HUNT4 study
Source: BMC Gastroenterol. 2026 Apr 16;26:330. doi: 10.1186/s12876-026-04837-y (PMC13224532; doi:10.1186/s12876-026-04837-y)
Supplement: Supplementary file 1 — Supplementary Material 1. [file 12876_2026_4837_MOESM1_ESM.docx]

# Supplementary Tables

Supplementary Table 1: Data dictionary of all variables

| **Category** | **New Variable** | **Old Variables** | **Old Levels** | **Recoding to New** | **Reference Category** |
| --- | --- | --- | --- | --- | --- |
| **Demography** | pid | pid_112437 |  | Retained as-is (ID; continuous) |  |
|  | sex | sex | 0=Female; 1=Male | factor: 1->Male, 0->Female; levels: Male, Female | Male |
|  | age | h4_partag_blm | Years | Retained (continuous) |  |
|  | inc | inc | 0=Controls; 1=Cases |  | Controls |
|  | education_cat | h4_educ_blq1 | 1,2,3,4,5,6 | 1-4 -> <13 years; 5-6 -> >=13 years | <13 years |
|  | municipality | h4_partmunicigeo_blm | 1=Inland; 2=Fjord; 3=Coast | Recode to labels; factor levels: Fjord, Inland, Coast | Fjord |
| **Genetic** | prs | scoresum | Numeric score | Rank-normalized (RankNorm) |  |
| **Lifestyle** | bmi_cat | h4_bmi_blm | Numeric BMI | <=18.4 Underweight; <=24.9 Normal; <=29.9 Overweight; >=30 Obesity; levels: Normal weight, Underweight, Overweight, Obesity | Normal weight |
|  | smoking_status_cat | h4_smostat_blq1 | 0=Never; 1-4=Ever | Recode to Never vs Ever; levels: Never, Ever | Never |
|  | exercise_freq_cat | h4_exef_blq1 | 1,2,3,4,5 | 1-3 -> Never-Moderate; 4-5 -> High; levels: Never-Moderate, High | Never-Moderate |
|  | alcohol_freq_cat | h4_alcfly_blq1 | 1,2,3,4,5,6 | 1 or 6 -> Never; 2-3 -> Moderate; 4-5 -> High; levels: Moderate, Never, High | Moderate |
|  | bread_total_cat | h4_whibreadn_blq2; h4_semiwhogrbreadn_blq2; h4_whogrbreadn_blq2; h4_extrawhogrbreadn_blq2 | Numeric frequency scores; all NA->NA | Row mean <3 -> <3 per day; >=3 -> >=3 per day; levels: <3 per day, >=3 per day | <3 per day |
| **Symptoms** | health_status_cat | h4_healt_blq1 | 1,2,3,4 | 1-2 -> Poor/Not good; 3-4 -> Good/Very good; levels: Good/Very good, Poor/Not good | Good/Very good |
|  | chronic_discomfort | h4_dischr_blq1 | 0=No; 1=Yes | Recode to No/Yes; levels: No, Yes | No |
|  | any_phy_pain_cat | h4_jopamor6wly_blq1; h4_mspachrl4w_blq1; h4_mspaly_blq2; h4_haly_blq2; h4_migev_blq1; h4_medmspaflm_blq1; h4_medhaflm_blq1 | Binary items 0/1; physical pain: 1=lowest; pain meds: 1=lowest | Yes if any: joint=1 OR physical>1 OR muscular=1 OR headache=1 OR migraine=1 OR pain_meds>1; No if joint=0 & physical=1 & muscular=0 & headache=0 & migraine=0; levels: No, Yes | No |
|  | mental_prob_cat | h4_tirecu_blq2; h4_mentprev_blq1; h4_mempr_blq2; h4_hadsdepr_blq2; h4_hadsanxi_blq2; h4_anxideprmedcu_blq1 | Fatigue, mental problems: 0/1; Memory: 0+; HADS-D/A numeric; Anx/Dep meds: 0+ | Yes if fatigue=1 OR mental=1 OR memory>0 OR HADS-D>7 OR HADS-A>7 OR meds>0; No if fatigue=0 & mental=0 & memory=0 & HADS-D<=7 & HADS-A<=7; levels: No, Yes | No |
|  | life_satisf_cat | h4_satlif_blq1 | 1,2,3,4,5,6,7 | 1-2 -> High; 3-7 -> Low-Moderate; levels: High, Low-Moderate | High |
|  | gi_complain_cat | h4_gipaly_blq2; h4_ginausly_blq2; h4_gidiarly_blq2; h4_giobstly_blq2; h4_gidefechaly_blq2; h4_gigasly_blq2; h4_gicardialy_blq2; h4_medesophflm_blq1; h4_medobstflm_blq1 | GI items lowest: stomach=0, others=1; GI meds: 1=lowest | Yes if any GI symptom/med above baseline (stomach>0 OR others>1 OR meds>1); Never if stomach=0 & others=1; levels: Never, Yes | Never |
| **Comorbidities** | cancer | h4_caev_blq1 | 0=No; 1=Yes | Recode to No/Yes; levels: No, Yes | No |
|  | cvd_cat | h4_caratrfibrev_blq1; h4_carinfev_blq1; h4_carfaiev_blq1; h4_carangev_blq1; h4_apoplev_blq1; h4_bpmedcu_blq1 | Each 0/1 | Yes if any(AF, MI, HF, angina, stroke)=1 or BP_meds=1; No if all AF=MI=HF=angina=stroke=0; levels: No, Yes | No |
|  | diabetes_cat | h4_diaev_blq1; h4_hypegluev_blq1; h4_blohba1cifcc_blm | Diabetes/hyperglycaemia: 0/1; HbA1c numeric (mmol/mol) | Yes if diabetes=1 OR hyperglycaemia=1 OR HbA1c>=48; No if diabetes=0 & hyperglycaemia=0 & HbA1c<48; levels: No, Yes | No |
|  | thyroid_cat | h4_thyhypoev_blq1; h4_thyhypeev_blq1; h4_thymedcu_blq1; h4_setsh_blm | Hypo/Hyper: 0/1; Thyroid meds: 0/1; TSH numeric | Yes if hypo=1 OR hyper=1 OR meds=1 OR TSH outside 0.5-3.6; No if hypo=0 & hyper=0 & 0.5<=TSH<=3.6; levels: No, Yes | No |
|  | ctd_cat | h4_rharthev_blq1; h4_sponarthev_blq1; h4_gouev_blq1 | Presence typically 0/1 | Yes if any >0; No if all 0; levels: No, Yes | No |
| **Biomarkers** | egfr_cat | h4_gfrestckd_blm | eGFR numeric (mL/min/1.73m2) | <90 -> Reduced; >=90 -> Normal; levels: Normal, Reduced | Normal |
|  | lipids_cat | h4_sechol_blm; h4_chollomedcu_blq1; h4_setrig_blm; h4_sehdlchol_blm; sex | Cholestrol, Triglydeceride numeric; HDL numeric (sex-specific); Cholestrol medication:0/1 | Abnormal if (chol>=6 or cholmed=1) OR TG>=1.7 OR (HDL<1.0 if Male, <1.3 if Female); Normal if all normal; levels: Normal, Abnormal | Normal |
|  | crp_cat | h4_secrp_blm | CRP numeric (mg/L) | <3 -> Normal; >=3 -> High; levels: Normal, High | Normal |
|  | hemoglobin_cat | h4_blohb_blm; sex | Hb numeric (g/dL); sex used for thresholds | Anemia if (Male<13.4; Female<11.7); else Normal; levels: Normal, Anemia | Normal |
| BMI: Body mass index; CVD: Cardiovascular disease; eGFR: Estimated glomerular filtration rate; CRP: C-reactive protein; GI: Gastrointestinal; HADS: Hospital anxiety and depression scale; Hb: Hemoglobin; HDL: High-density lipoprotein; INC: Incidence case; PRS: Polygenic risk score; TSH: Thyroid stimulating hormone | | | | | |
| Physical pain included joint pain, muscular pain, headache, including migraine, and medicine for physical pain; Mental problems included fatigue, anxiety, depression, memory problems, symptoms of anxiety or depression, and medicine for anxiety or depression; Gastrointestinal complaints included stomach pain, nausea, diarrhea, constipation, bloating, heartburn, and medication for heartburn and constipation; Cardiovascular disease included self-reported atrial fibrillation, myocardial infarctions, heart failure, angina, high blood pressure, stroke, and medicine for high blood pressure; Diabetes mellitus included self-reported diabetes status and hyperglycemia and high HbA1c values; Thyroid disease included self-reported hypothyroidism, hyperthyroidism, medication for thyroid disease, and pathological TSH values; Connected tissue disease included self-reported rheumatoid arthritis, gout, and Bechterew disease. | | | | | |

Supplementary Table 2: Penalized and unpenalized estimates for the dummy encoded selected variables from regression

| **Variable** | **Penalized Beta** | **Unpenalized Beta** | **Unpenalized SE** | **p value** | **Adjusted OR (95% CI)** |
| --- | --- | --- | --- | --- | --- |
| Chronic discomfort_Yes | -0.090 | -0.249 | 0.135 | 0.065 | 0.779 (0.598-1.016) |
| Diabetes_Yes | -0.038 | -0.638 | 0.269 | 0.018 | 0.529 (0.312-0.896) |
| eGFR_Reduced | -0.142 | -0.397 | 0.123 | 0.001 | 0.673 (0.528-0.856) |
| Health status_Poor/NotGood | -0.101 | -0.378 | 0.177 | 0.033 | 0.685 (0.484-0.970) |
| PRS | 1.065 | 1.275 | 0.060 | <0.001 | 3.578 (3.180-4.025) |

Supplementary Table 3: Distribution of cases and non-cases in the various cross-validation folds of the training dataset, and test dataset.

| **Dataset** | **Fold Type** | **Outer Fold** | **Inner Fold** | **Total^1^** | **Cases^1^** | **Controls^1^** | **Prevalence^2^** |
| --- | --- | --- | --- | --- | --- | --- | --- |
| Training | Outer_Validation | 1 | NA | 12129 | 125 | 12004 | 1.03% |
| Training | Outer_Training | 1 | NA | 24257 | 194 | 24063 | 0.8% |
| Training | Inner_Validation | 1 | 1 | 8085 | 70 | 8015 | 0.87% |
| Training | Inner_Training | 1 | 1 | 16172 | 124 | 16048 | 0.77% |
| Training | Inner_Validation | 1 | 2 | 8086 | 59 | 8027 | 0.73% |
| Training | Inner_Training | 1 | 2 | 16171 | 135 | 16036 | 0.83% |
| Training | Inner_Validation | 1 | 3 | 8086 | 65 | 8021 | 0.8% |
| Training | Inner_Training | 1 | 3 | 16171 | 129 | 16042 | 0.8% |
| Training | Outer_Validation | 2 | NA | 12129 | 107 | 12022 | 0.88% |
| Training | Outer_Training | 2 | NA | 24257 | 212 | 24045 | 0.87% |
| Training | Inner_Validation | 2 | 1 | 8086 | 68 | 8018 | 0.84% |
| Training | Inner_Training | 2 | 1 | 16171 | 144 | 16027 | 0.89% |
| Training | Inner_Validation | 2 | 2 | 8086 | 69 | 8017 | 0.85% |
| Training | Inner_Training | 2 | 2 | 16171 | 143 | 16028 | 0.88% |
| Training | Inner_Validation | 2 | 3 | 8085 | 75 | 8010 | 0.93% |
| Training | Inner_Training | 2 | 3 | 16172 | 137 | 16035 | 0.85% |
| Training | Outer_Validation | 3 | NA | 12128 | 87 | 12041 | 0.72% |
| Training | Outer_Training | 3 | NA | 24258 | 232 | 24026 | 0.96% |
| Training | Inner_Validation | 3 | 1 | 8086 | 85 | 8001 | 1.05% |
| Training | Inner_Training | 3 | 1 | 16172 | 147 | 16025 | 0.91% |
| Training | Inner_Validation | 3 | 2 | 8086 | 72 | 8014 | 0.89% |
| Training | Inner_Training | 3 | 2 | 16172 | 160 | 16012 | 0.99% |
| Training | Inner_Validation | 3 | 3 | 8086 | 75 | 8011 | 0.93% |
| Training | Inner_Training | 3 | 3 | 16172 | 157 | 16015 | 0.97% |
| Test | Test_Set | NA | NA | 15594 | 146 | 15448 | 0.94% |
| Training | Full_Training_Set | NA | NA | 36386 | 319 | 36067 | 0.88% |
| ^1^ indicates the number of individuals, N  ^2^ indicates the percentage of individuals, %  NA indicates not applicable | | | | | | | |

Supplementary Table 4: Sensitivity analysis at different prevalence thresholds

| **Prevalence** | **AUROC** | **PRAUC** | **Threshold** | **Sensitivity** | **Specificity** | **PPV** | **NPV** |
| --- | --- | --- | --- | --- | --- | --- | --- |
| 1.0% | 0.816 (0.780-0.849) | 0.048 (0.037-0.067) | 0.002 | 0.973 (0.945-0.993) | 0.140 (0.134-0.146) | 0.011 (0.011-0.012) | 0.998 (0.996-1.000) |
|  |  |  | 0.004 | 0.925 (0.877-0.966) | 0.447 (0.439-0.455) | 0.017 (0.016-0.017) | 0.998 (0.997-0.999) |
|  |  |  | 0.007 | 0.863 (0.801-0.918) | 0.608 (0.600-0.616) | 0.022 (0.020-0.023) | 0.998 (0.997-0.999) |
|  |  |  | 0.032 | 0.240 (0.171-0.308) | 0.958 (0.955-0.961) | 0.054 (0.040-0.069) | 0.992 (0.991-0.993) |
|  |  |  | 0.167 | 0.000 (0.000-0.000) | 1.000 (0.999-1.000) | 0.000 (0.000-0.000) | 0.990 (0.990-0.990) |
| 2.5% | 0.816 (0.779-0.851) | 0.111 (0.087-0.150) | 0.002 | 0.973 (0.945-0.993) | 0.138 (0.129-0.147) | 0.028 (0.027-0.029) | 0.995 (0.990-0.999) |
|  |  |  | 0.004 | 0.925 (0.884-0.966) | 0.442 (0.429-0.454) | 0.041 (0.039-0.043) | 0.996 (0.993-0.998) |
|  |  |  | 0.007 | 0.863 (0.808-0.918) | 0.606 (0.593-0.618) | 0.053 (0.050-0.057) | 0.994 (0.992-0.997) |
|  |  |  | 0.032 | 0.240 (0.171-0.308) | 0.959 (0.954-0.964) | 0.131 (0.096-0.168) | 0.980 (0.978-0.982) |
|  |  |  | 0.167 | 0.000 (0.000-0.000) | 1.000 (0.999-1.000) | 0.000 (0.000-0.000) | 0.975 (0.975-0.975) |
| 5.0% | 0.816 (0.777-0.850) | 0.217 (0.169-0.276) | 0.002 | 0.973 (0.945-0.993) | 0.141 (0.128-0.155) | 0.056 (0.054-0.058) | 0.990 (0.979-0.998) |
|  |  |  | 0.004 | 0.925 (0.877-0.966) | 0.444 (0.426-0.463) | 0.081 (0.076-0.085) | 0.991 (0.986-0.996) |
|  |  |  | 0.007 | 0.863 (0.801-0.918) | 0.601 (0.583-0.620) | 0.102 (0.095-0.109) | 0.988 (0.983-0.993) |
|  |  |  | 0.032 | 0.240 (0.171-0.308) | 0.961 (0.953-0.968) | 0.243 (0.181-0.308) | 0.960 (0.957-0.964) |
|  |  |  | 0.167 | 0.000 (0.000-0.000) | 1.000 (1.000-1.000) | NA | 0.950 (0.950-0.950) |
| 10.0% | 0.818 (0.778-0.853) | 0.352 (0.287-0.426) | 0.002 | 0.973 (0.945-0.993) | 0.151 (0.132-0.170) | 0.113 (0.109-0.116) | 0.980 (0.959-0.995) |
|  |  |  | 0.004 | 0.925 (0.877-0.966) | 0.461 (0.436-0.488) | 0.160 (0.150-0.169) | 0.982 (0.971-0.991) |
|  |  |  | 0.007 | 0.863 (0.801-0.918) | 0.618 (0.594-0.645) | 0.201 (0.186-0.217) | 0.976 (0.966-0.985) |
|  |  |  | 0.032 | 0.240 (0.171-0.308) | 0.960 (0.951-0.970) | 0.402 (0.310-0.500) | 0.919 (0.912-0.926) |
|  |  |  | 0.167 | 0.000 (0.000-0.000) | 1.000 (1.000-1.000) | NA | 0.900 (0.900-0.900) |
