# Supplementary material for: A model for predicting celiac disease among undiagnosed subjects established with data from the HUNT4 study
Source: BMC Gastroenterol. 2026 Apr 16;26:330. doi: 10.1186/s12876-026-04837-y (PMC13224532; doi:10.1186/s12876-026-04837-y)

# Supplementary Figures


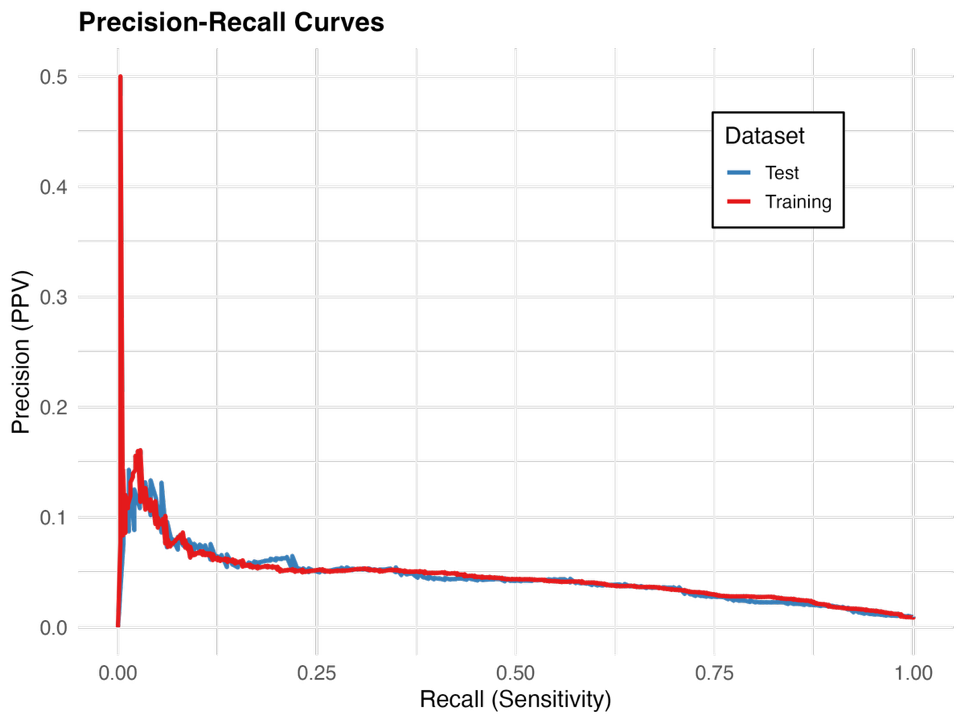


Supplementary Figure 1: Precision-Recall curve for the training and testing datasets


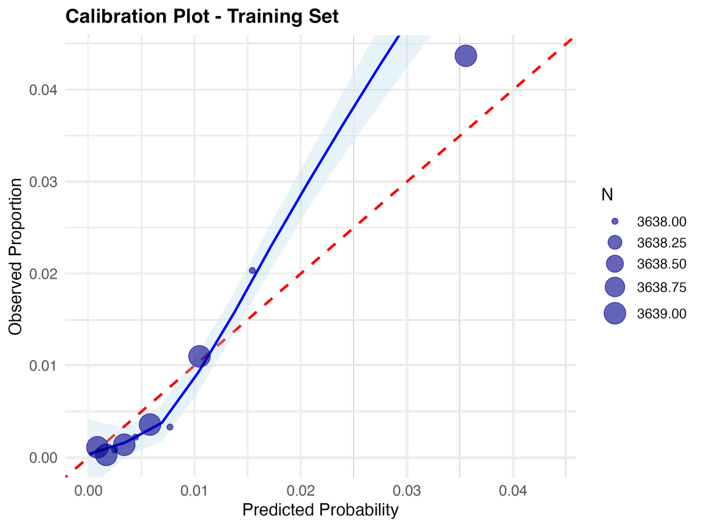

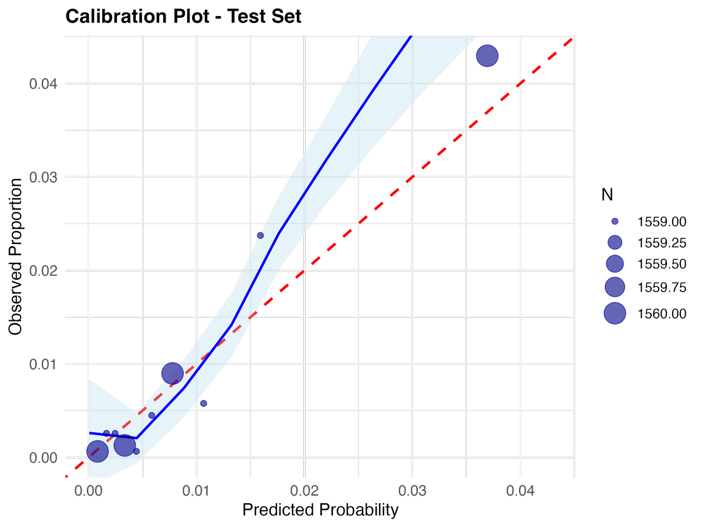


Supplementary Figure 2: Calibration plot for training and testing datasets

Supplementary Figure 3: Decision Curve Sensitivity Analysis at different prevalence levels.


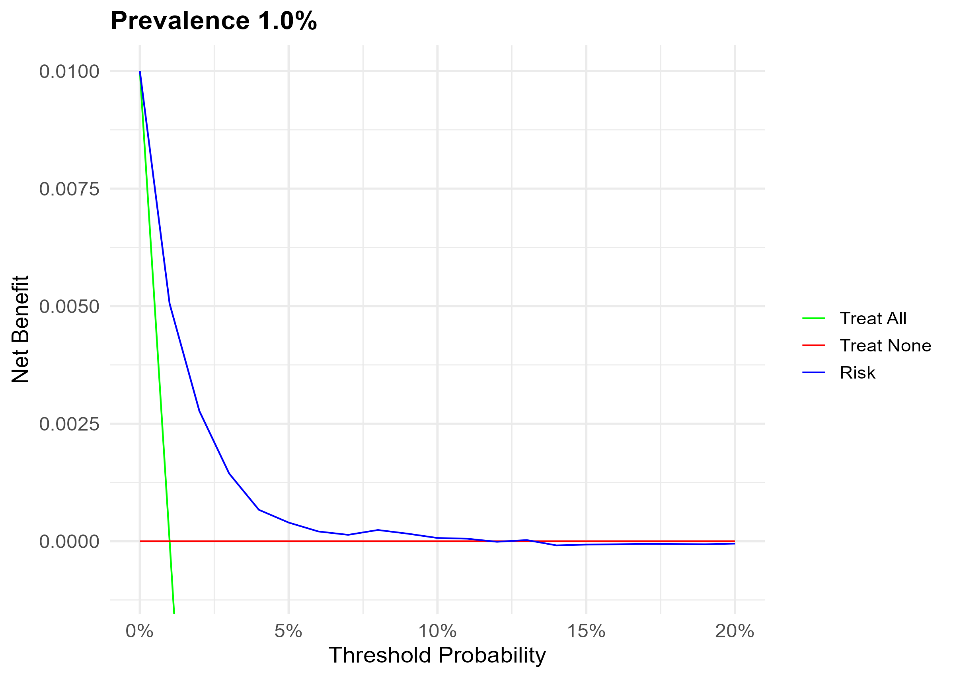

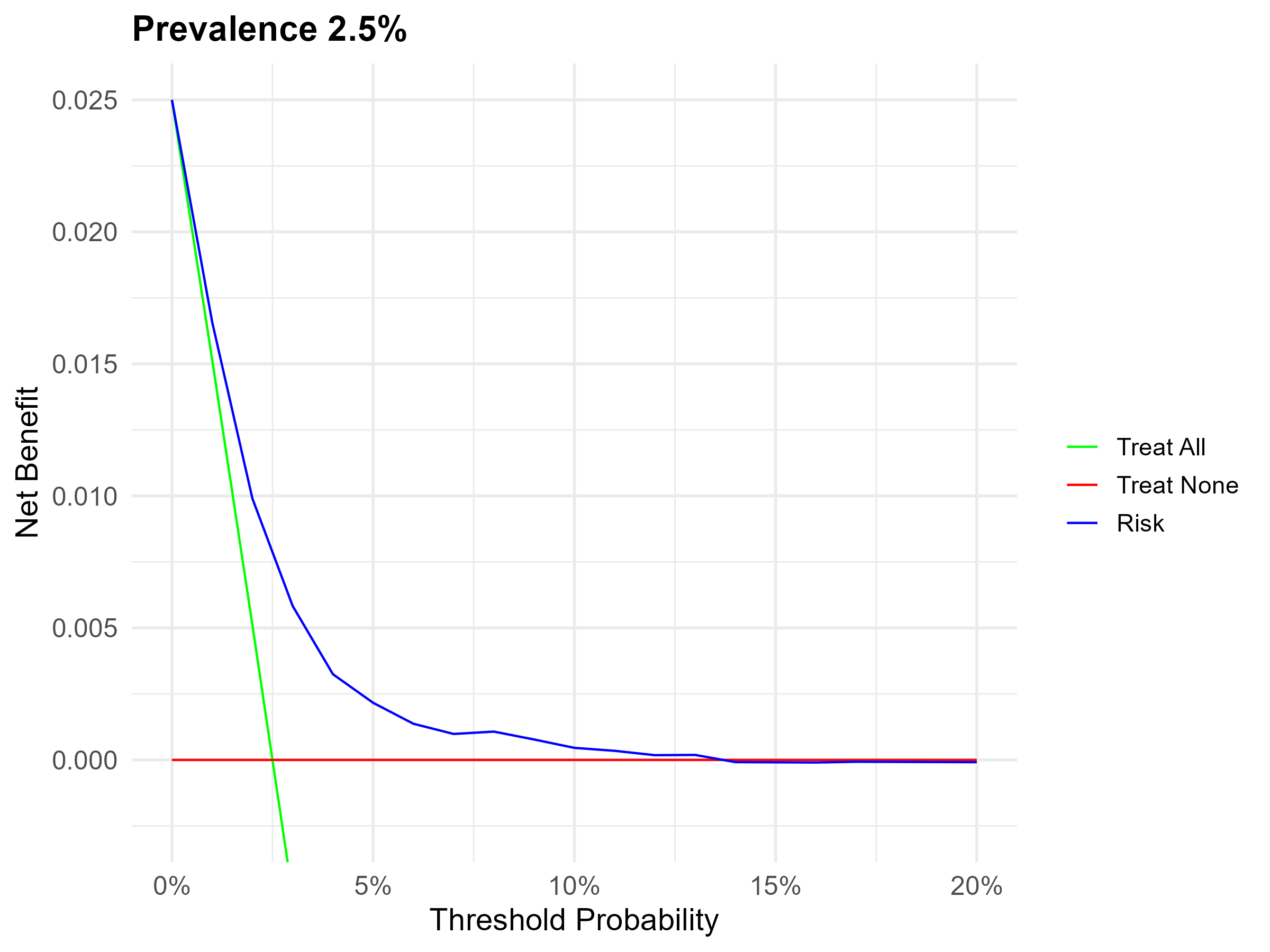

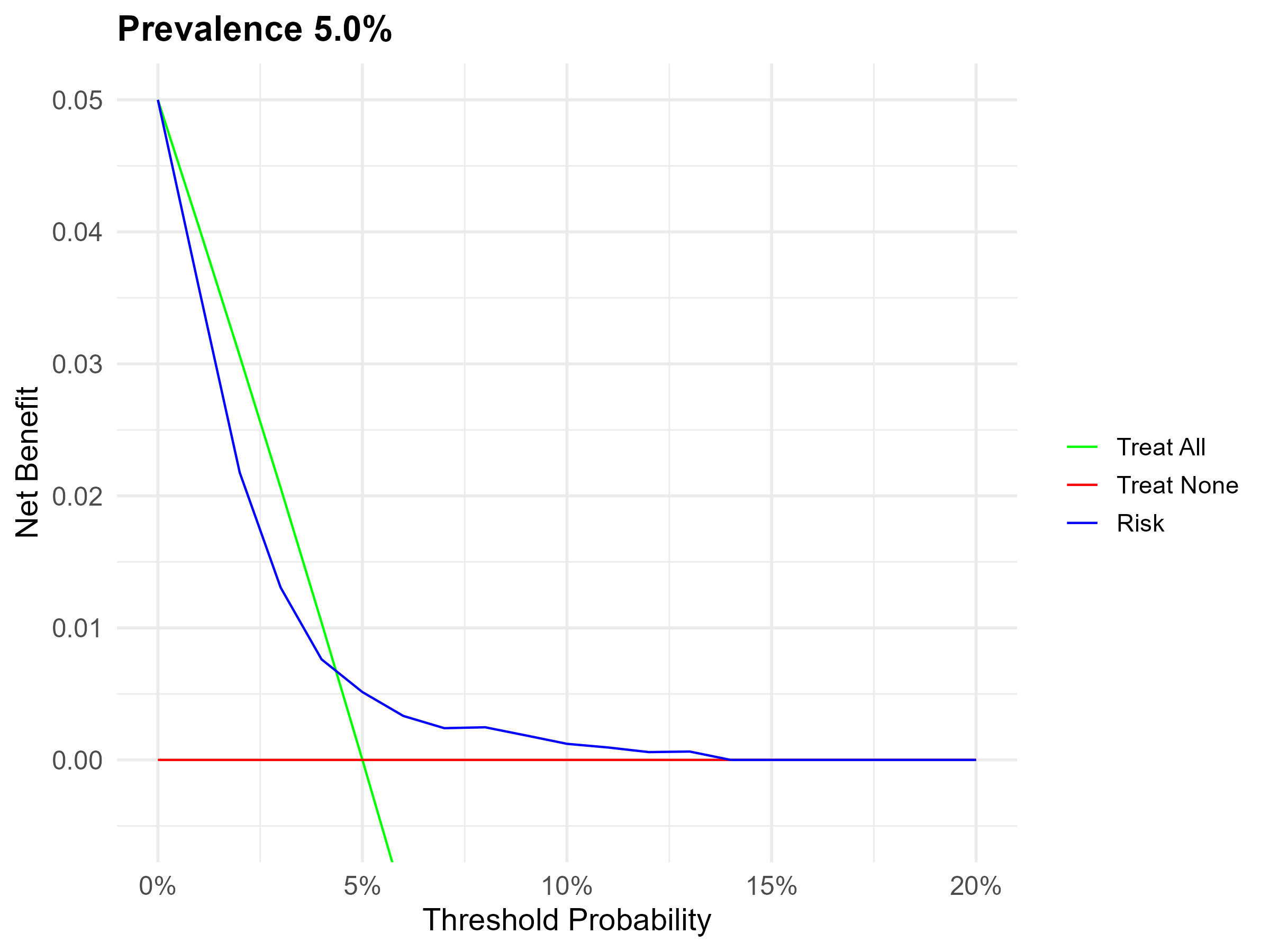

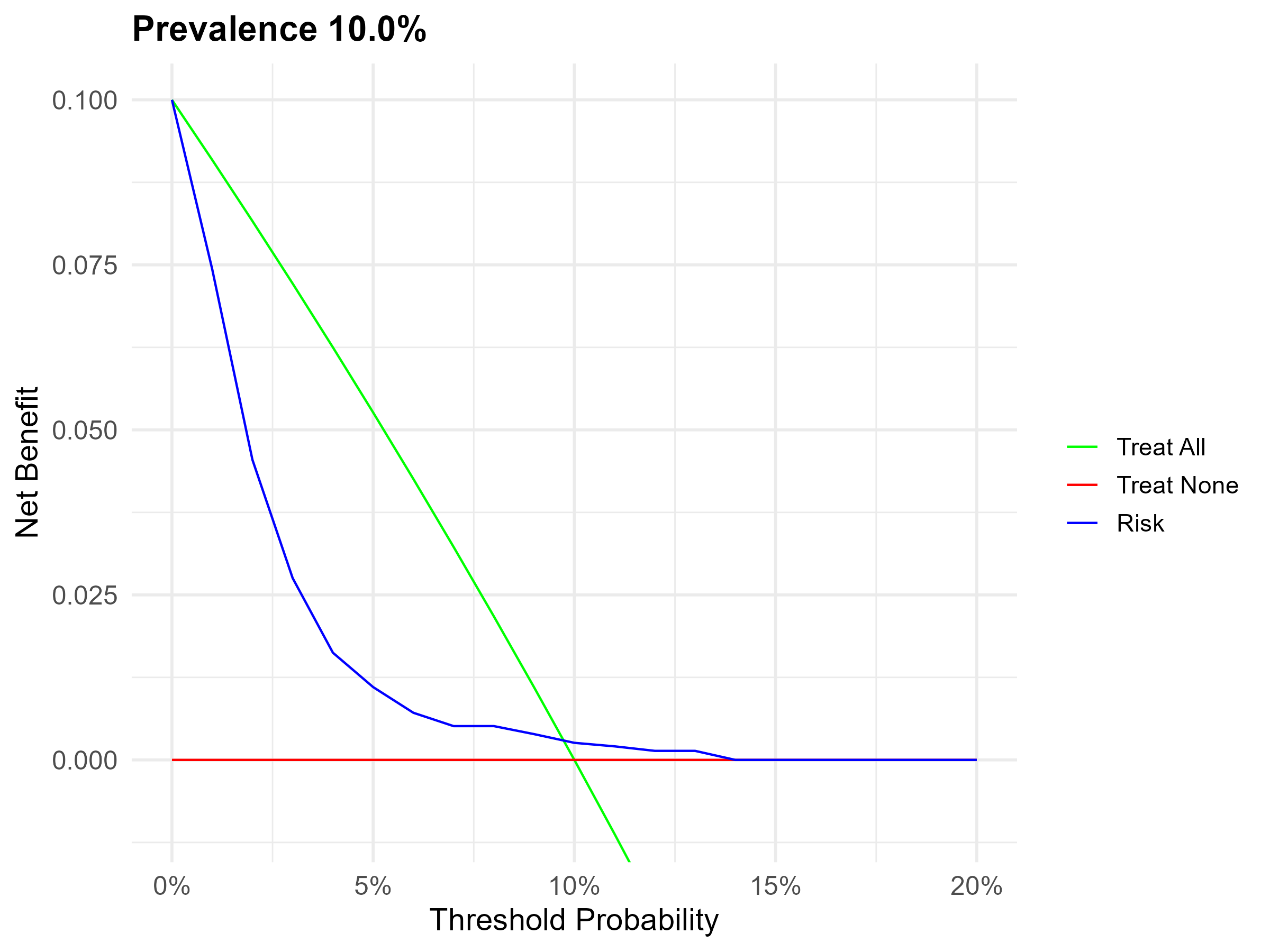

Supplement: Supplementary file 2 — Supplementary Material 2. [file 12876_2026_4837_MOESM2_ESM.docx]
